# Correlation between the Lunar Phase and Tail-Lifting Behavior of Lizards (*Pogona vitticeps*) Exposed to an Extremely Low-Frequency Electromagnetic Field

**DOI:** 10.3390/ani9050208

**Published:** 2019-04-30

**Authors:** Tsutomu Nishimura, Harue Tada, Masanori Fukushima

**Affiliations:** 1 Institute for Advancement of Clinical and Translational Science (iACT), Graduate School of Medicine, Kyoto University, 54 Kawahara-cho, Shogoin, Sakyo-ku, Kyoto 606-8507, Japan; haru.ta@kuhp.kyoto-u.ac.jp; 2 Translational Research Center for Medical Innovation, 1-5-4 Minatojima-minamimachi, Chuo-ku, Kobe 650-0047, Japan; mfukushi@tri-kobe.org

**Keywords:** lizards, magnetic sense, extremely low-frequency electromagnetic field, tail lifting, full moon, lunar phase

## Abstract

**Simple Summary:**

We examined the relationship between the number of tail lifts of lizards (*Pogona vitticeps*) and environmental factors, such as the calendar month, daily mean temperature, daily mean humidity, daily mean atmospheric pressure, lunar phase (full moon/new moon), and K index as a geomagnetic disturbance index using 16 months of data. We set up an extremely low-frequency electromagnetic field (ELF-EMF) group and a control group. In a multiple linear regression analysis, the independent determinants associated with the number of tail lifts were the full moon, the temperature, February, March, April, and May in the ELF-EMF group and March, April, May, and June in the control group. The *P. vitticeps* in the ELF-EMF group responded to the full moon whereas those in the control group did not.

**Abstract:**

We previously showed that the agamid lizard *Pogona vitticeps* responded to an extremely low-frequency electromagnetic field (ELF-EMF; frequency: 6 and 8 Hz; peak magnetic field: 2.6 µT; peak electric field: 10 V/m) with tail-lifting behavior. In addition, the tail-lifting response to ELF-EMF disappeared when the parietal eyes of the lizards were covered by small round aluminum caps. This result suggests that the parietal eye contributes to light-dependent magnetoreception. In the present study, we set up an ELF-EMF group to evaluate the long-term effect of the ELF-EMF on lizards’ behavior and examine our hypothesis that exposure to ELF-EMFs increases the magnetic field sensitivity in lizards. We therefore include the lunar phase (full moon/new moon) and K index as environmental factors related to the geomagnetic field in the analysis. The number of tail lifts per individual per day was the response variable while calendar month, daily mean temperature, daily mean humidity, daily mean atmospheric pressure, full moon, new moon, and K index were the explanatory variables. We analyzed an ELF-EMF group and a control group separately. In a multiple linear regression analysis, the independent determinants associated with the number of tail lifts were the full moon, the temperature, February, March, April, and May in the ELF-EMF group and March, April, May, and June in the control group. The *P. vitticeps* in the ELF-EMF group responded to the full moon whereas those in the control group did not. In addition, in the ELF-EMF group, the number of tail lifts was higher on days when the K index was higher (*P* = 0.07) in the first period whereas there was no such tendency in either period in the control group. There is the possibility that the exposure to ELF-EMFs may increase magnetic-field sensitivity in lizards.

## 1. Introduction

A wide range of taxa, such as mollusks, crustaceans, insects, fishes, birds, mammals, and amphibians, have a magnetic sense and use magnetic compasses to orient themselves in what is referred to as magnetoreception in animals [1]. Among reptiles, box turtles [2], leatherback sea turtles [3], green sea turtles [4,5], and loggerhead sea turtles [6,7] are thought to migrate using the earth’s geomagnetic field [8]. There are two hypotheses for magnetoreception. One is that magnetoreception employs a chemical compass based on a radical pair mechanism and the other is that magnetoreception has a magnetite-based mechanism [9,10].

We previously showed that the agamid lizard *Pogona vitticeps* responded to an extremely low-frequency electromagnetic field (ELF-EMF; 6 and 8 Hz; peak magnetic field, 2.6 µT; peak electric field, 10 V/m) with tail-lifting behavior [11]. In addition, the tail-lifting response to ELF-EMF disappeared when the lizards’ parietal eyes were covered by small round aluminum caps. This result suggests that the parietal eye is involved in light-dependent magnetoreception.

Lizard *Leiocephalus carinatus* shows tail-curing behavior during agonistic courtship [12] and the mating season of *P. vitticeps* occurs in spring [13]. The present paper therefore investigates the relationship between the number of tail lifts and season-related environmental factors, namely the calendar month, daily mean temperature, daily mean humidity, and daily mean atmospheric pressure.

Paddlefish (*Polyodon spathula*) use passive electroreceptors for the detection of electrical signals from planktonic prey [14]. Russell et al. reported that the normal feeding behavior of them was enhanced by stochastic resonance [14]. Russell et al. also demonstrated widening the spatial range for plankton detection when a noisy electric field of optimal amplitude is applied in water [14]. We set up an ELF-EMF group in the present study to evaluate the long-term effect of the ELF-EMF on lizards’ behavior and thus examine our hypothesis that exposure to ELF-EMFs increases the magnetic field sensitivity in lizards. We therefore include the lunar phase (full moon/new moon) and K index as environmental factors related to the geomagnetic field in the analysis. The K indices, devised by Bartels et al., provide an objective method of monitoring the effects of the solar wind and interplanetary magnetic field effects on the magnetic field of the earth [15]. The basis of the K indices is the range of irregular variations in geomagnetic fields, which are measured for the two horizontal geomagnetic components, after eliminating so-called non-K variations [15]. Regarding the mechanism for sensing the lunar phase, it was reported that there is a fluctuation in geomagnetism due to the lunar phase [16,17,18].

## 2. Materials and Methods

Central bearded dragons (*Pogona vitticeps* Ahl; Agamidae) were purchased from a commercial source (Daiwa Pet Co., Kyoto, Japan). Animal experiment protocols were approved by the Kyoto University Animal Research Committee (MedKyo09131).

K index values were obtained from the website of the Magnetic Observatory, Japan Meteorological Agency (http://www.kakioka-jma.go.jp).

### 2.1. First Period (8 Months)

Two adult males and four females (mean body mass: 207.5 ± 28.0 g; mean snout–vent length: 16.3 ± 1.3 cm; mean total length: 34.8 ± 4.6 cm) were used in this experiment. Central bearded dragons live in harem groups in the wild. The lizards were therefore divided randomly into two groups of three lizards each (the EMF and control groups) such that each group had one male and two females. In this experiment, we kept lizards of the same group together in the same terrarium (length × width × height: 90 × 45 × 45 cm) but in a terrarium separate from that of lizards of the other group. The terrariums were made of glass and resin. The two terrariums were in the same room. The paired coils produced a sinusoidal 6 and 8 Hz EMF with a peak magnetic field of 2.6 µT and a peak electric field of 10 V m^−1^.The terrariums were subject to a light/dark cycle of 14 h:10 h produced with an ultraviolet light (≈15,000 lux; lights on at 08:00) and an incandescent light bulb (≈6000 lux; lights on at 08:00).

The EMF group received whole-body exposure to an ELF-EMF for 12 h per day (09:00–21:00). The EMF group was subjected to an ELF-EMF during the light period.

The method of ELF-EMF exposure, monitoring of the number of tail lifts, and defining of tail lifts were the same as those in a previous study [11]. The first period (8 months) of the experiment lasted from 27 April 2007 to 18 December 2007.

### 2.2. Second Period (16 Months)

We did not control the air temperature or humidity but began recording the air temperature, humidity, and atmospheric pressure every hour for 24 h per day on 19 December 2007. We added one female lizard to each group such that each group had one male and three females on 19 December 2007. The method of ELF-EMF exposure, monitoring of the number of tail lifts, and defining of tail lifts were the same as those described for the first period (8 months). The second period (16 months) of the experiment lasted from 19 December 2007 to 30 April 2009.

### 2.3. Statistics

We investigated the relationship between the number of tail lifts and environmental factors, such as the calendar month, lunar phase (full moon/new moon), and K index, through multiple linear regression analyses. Analysis of the data for the first 8 months (i.e., the first period) took the number of tail lifts per individual per day as the response variable and the calendar month, full moon, new moon, and K index as explanatory variables. Analysis of the data for the following 16 months (i.e., the second period) took the number of tail lifts per individual per day as the response variable and the calendar month, full moon, new moon, K index, daily mean temperature, daily mean humidity, and daily mean atmospheric pressure as explanatory variables. We used full moon and new moon variables as binary variables for analysis. We defined 0 and 1 based on the lunar phase calendar. The factors were inserted into the multiple regression model. We analyzed the ELF-EMF group and control group separately. A value of 0.05 indicated statistical significance. Statistical analyses were performed using SAS ver. 9.2 (SAS Institute Inc., Cary, NC, USA).

## 3. Results

### 3.1. Overall 24 Months

**Figure 1:**
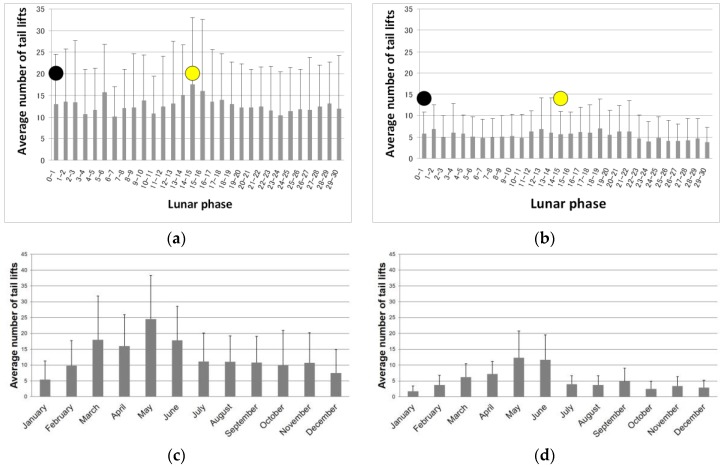
(**a**) Average number (+standard deviation (SD)) of tail lifts by moon phase in the ELF-EMF group. The full moon falls on either the 14th or 15th day of the lunar phase (yellow stamp) while the new moon falls on the zeroth day of the lunar phase (black stamp). (**b**) Average number (+SD) of tail lifts by moon phase in the control group. The full moon falls on either the 14th or 15th day of the lunar phase while the new moon falls on the zeroth day of the lunar phase. (**c**) Average number (+SD) of tail lifts by month in the ELF-EMF group. (**d**) Average number (+SD) of tail lifts by month in the control group.

The average numbers of tail lifts by lunar phase are shown in Figure 1a,b for the ELF-EMF and control groups. The average numbers of tail lifts by month are shown in Figure 1c,d for the ELF-EMF and control groups.

### 3.2. First Period (8 Months)

In multiple linear regression analysis using the first 8 months of data, the independent determinants associated with the number of tail lifts were the full moon, May, and June in the ELF-EMF group, as shown in Table 1, and April, May, June, and July in the control group, as shown in Table 2. In the relevance to the K index, there was a tendency (*P* = 0.07), but not significant in the ELF-EMF group.

### 3.3. Second Period (16 Months)

In multiple linear regression analysis using the second 16 months of data, the independent determinants associated with the number of tail lifts were the full moon, daily mean temperature, February, March, April, and May in the ELF-EMF group, as shown in Table 1, and March, April, May, and June in the control group, as shown in Table 2.

## 4. Discussion

March, April, and May were positively correlated with the number of tail lifts in both the ELF-EMF and control groups. This result agrees with the results of previous reports that the mating season of *P. vitticeps* occurs in spring [13] and that the lizard *Leiocephalus carinatus* curls its tail as a component of display during courtship [12].

In the ELF-EMF group, the number of tail lifts was higher on days when the K index was higher (*P* = 0.07) in the first period whereas there was no such tendency in either period in the control group. Because this tendency was observed only in the ELF-EMF group, there is the possibility that the exposure to ELF-EMFs may increase magnetic-field sensitivity in lizards.

The *P. vitticeps* in the ELF-EMF group responded to the full moon whereas those in the control group did not. Although it has been reported that the activity of the nocturnal gecko increases at full moon [19], the relevant experiment was conducted outdoors, and the nocturnal gecko could therefore see the full moon, whereas there was no window in the experiment room in the present study such that the *P. vitticeps* in the ELF-EMF group responded only to environmental changes related with the full moon. Periodicity of the geomagnetic fields by lunar phase has been reported [16,17,18] and the lizards may have responded to the full moon by sensing changes in the geomagnetic field.

It has been reported that the reproductive cycles of fish, birds, amphibians, and mammals correlate with the lunar phase [20,21,22,23,24]. In terms of an evolutionary meaning, fish may have more success when producing eggs at a time that other animals are also in a reproductive condition [25]. Regarding the mechanism for sensing the lunar phase, it is noted that there is a fluctuation in geomagnetism due to the lunar phase [16,17,18]. Grant et al. reported that newts use gravitational and/or geomagnetic changes related to the lunar cycle to time their arrivals [24]. Tail-lifting and reproductive cycles are different behaviors, but both may be actions whereby the full moon is sensed according to geomagnetic fluctuations.

A limitation of our study was the group rearing of the lizards. However, Kircher and Johnson reported that tail curling displays are not a necessary component of social interactions [26]. It is therefore thought that the result would not change between group rearing and individual rearing.

The present study found a significant difference in behavior between *P. vitticeps* exposed to an ELF-EMF and those not exposed, with there being a correlation between the tail-lifting frequency of exposed animals and the month of the year corresponding to the known mating season, as well as between the tail-lifting frequency and full moon. In addition, in the ELF-EMF group, the number of tail lifts was higher on days when the K index was higher (*P* = 0.07) in the first period whereas there was no such tendency in either period in the control group. There is the possibility that the exposure to ELF-EMFs may increase magnetic-field sensitivity in lizards. These results require further investigation even though explanatory mechanisms have been suggested by other studies.

## Figures and Tables

**Table 1:** Results of a multiple linear regression analysis investigating the number of tail lifts against the calendar month, full moon, new moon, K index, daily mean temperature, daily mean humidity, and daily mean atmospheric pressure in the ELF-EMF group.

Variable	First Period (8 Months)			Second Period (16 Months)		
	Coefficient (B)	SE *	*P*	Coefficient (B)	SE	*P*
Full moon	6.9	3.1	0.03	5.5	2.5	0.03
New moon	4.2	3.1	0.18	0.8	2.5	0.76
Daily mean temperature	–	–	–	1.20	0.27	<0.0001
Daily mean humidity	–	–	–	0.08	0.08	0.33
Daily mean atmospheric pressure	–	–	–	−0.04	0.10	0.73
K index	0.15	0.09	0.07	−0.02	0.08	0.78
January	–	–	–	−0.3	2.0	0.88
February	–	–	–	4.4	2.1	0.04
March	–	–	–	11.1	2.1	<0.0001
April	9.3	4.9	0.06	4.9	2.4	0.04
May	12.3	2.6	<0.0001	10.3	3.2	0.001
June	6.5	2.6	0.01	0.2	3.4	0.94
July	−1.4	2.6	0.60	−6.0	3.6	0.09
August	−1.3	2.6	0.62	−5.6	3.5	0.11
September	0.1	2.6	0.98	−4.9	3.2	0.12
October	−1.8	2.6	0.48	1.3	2.8	0.65
November	1.8	2.6	0.50	2.2	2.4	0.38
December	Reference			Reference		

* SE: standard error.

**Table 2:** Results of a multiple linear regression analysis investigating the number of tail lifts against the calendar month, full moon, new moon, K index, daily mean temperature, daily mean humidity, and daily mean atmospheric pressure in the control group.

Variable	First Period (8 Months)			Second Period (16 Months)		
	Coefficient (B)	SE *	*P*	Coefficient (B)	SE	*P*
Full moon	0.7	1.0	0.52	0.2	1.0	0.86
New moon	−0.1	1.0	0.93	−0.3	1.0	0.75
Daily mean temperature	–	–	–	0.20	0.11	0.06
Daily mean humidity	–	–	–	−0.04	0.03	0.21
Daily mean atmospheric pressure	–	–	–	−0.07	0.04	0.08
K index	−0.01	0.03	0.76	−0.02	0.03	0.43
January	–	–	–	−1.5	0.8	0.07
February	–	–	–	0.2	0.8	0.78
March	–	–	–	2.4	0.8	0.003
April	9.5	1.6	<0.0001	2.3	0.9	0.01
May	4.9	0.9	<0.0001	12.8	1.3	<0.0001
June	4.8	0.9	<0.0001	11.6	1.3	<0.0001
July	2.2	0.9	0.01	−1.6	1.4	0.27
August	1.6	0.9	0.07	−1.3	1.4	0.33
September	1.2	0.9	0.17	2.3	1.3	0.06
October	−0.1	0.9	0.89	0.2	1.1	0.84
November	0.8	0.9	0.38	0.8	1.0	0.40
December	Reference			Reference		

* SE: standard error.
